# Comparing the diagnostic considerations between general practitioners with a special interest in cardiovascular disease and those without in patients with symptoms suggestive of heart failure: a vignette study

**DOI:** 10.1186/s12875-024-02466-6

**Published:** 2024-06-14

**Authors:** Cornelia J. C. Vermeer, Amy Groenewegen, Monika Hollander, Janneke Schuring, Ingrid Looijmans–van den Akker, Andrew Oostindjer, Huug van Duijn, Ineke Nederend, Frans H. Rutten

**Affiliations:** 1 Department of General Practice & Nursing Science, Julius Centre for Health Sciences and Primary Care, University Medical Centre Utrecht, Utrecht University, Utrecht, The Netherlands; 2 Leidsche Rijn Julius Healthcare Centers, Utrecht, The Netherlands; 3 General Practice Oostindjer, Oldenzaal, The Netherlands; 4 Care Group Katwijk, Katwijk, The Netherlands; 5 Dutch Heart Foundation, The Hague, The Netherlands

**Keywords:** Heart failure, Diagnosis, Clinical decision-making, General practice, Primary health care

## Abstract

**Background:**

General practitioners (GPs) specialized in cardiovascular disease (GPSI-CVD) may suspect heart failure (HF) more easily than GPs not specialized in CVD. We assessed whether GPSI-CVD consider investigations aimed at detecting HF more often than other GPs in two clinical scenarios of an older male person with respiratory and suggestive HF symptoms.

**Methods:**

In this vignette study, Dutch GPs evaluated two vignettes. The first involved a 72-year-old man with hypertension and a 30 pack-year smoking history who presented himself with symptoms of a common cold, but also shortness of breath, reduced exercise tolerance, and signs of fluid overload. The second vignette was similar but now the 72-year-old man was known with chronic obstructive pulmonary disease (COPD).

GPs could select diagnostic tests from a multiple-choice list with answer options targeted at HF, COPD or exacerbation of COPD, or lower respiratory tract infection. With Pearson Chi-square or Fisher’s exact test differences between the two GP groups were assessed regarding the chosen diagnostic tests.

**Results:**

Of the 148 participating GPs, 25 were GPSI-CVD and 123 were other GPs. In the first vignette, GPSI-CVD more often considered performing electrocardiography (ECG) than other GPs (64.0% vs. 32.5%, *p* = 0.003). In the second vignette, GPSI-CVD were more inclined to perform both ECG (36.0% vs. 12.2%, *p* = 0.003) and natriuretic peptide testing (56.0% vs. 32.5%, *p *= 0.006).

**Conclusions:**

Most GPs seemed to consider multiple diagnoses, including HF, with GPSI-CVD more likely performing ECG and natriuretic peptide testing in an older male person with both respiratory and suggestive HF symptoms.

**Supplementary Information:**

The online version contains supplementary material available at 10.1186/s12875-024-02466-6.

## Introduction

Heart failure (HF) is a clinical syndrome characterized by breathlessness, reduced exercise tolerance/fatigue often accompanied by signs of fluid overload (e.g. elevated jugular venous pressure, pulmonary crackles, peripheral oedema) and a broadened/sustained apical impulse in left lateral decubital position [1]. In the Netherlands, around 240,000 patients are diagnosed with HF^2^, while another 255,000 is yet unrecognized [2]. Thus, underdiagnosis of HF is very common, especially in those with preserved ejection fraction [3, 4]. Conditions such as chronic obstructive pulmonary disease (COPD) can mimic HF symptoms, but COPD patients also have an increased risk of developing HF [5–8]. Finally, pulmonary fluid caused by HF may result in external bronchial obstruction and thus obstruction on spirometry, which can cause misclassification of these patients as COPD [6].

Evidently, the assessment of shortness of breath, prevalent in both HF and COPD, is challenging for general practitioners (GPs). While tests like electrocardiography (ECG), natriuretic peptide testing and spirometry are accessible in primary care, for echocardiography typically referral to the cardiologist is necessary.

(Inter)national guidelines on HF recommend referral for echocardiography in individuals with symptoms and/or signs suggestive of HF if natriuretic peptide levels are above the exclusionary threshold and/or patients have an abnormal electrocardiogram [1, 9]. Previous studies have shown that adherence of GPs to these HF guidelines is not optimal [10, 11]. This may result in over- or underdiagnosis of HF, and especially misclassification as COPD is common [6, 8, 12].

In the Netherlands, there are around 80 GPs specialized in cardiovascular disease (GPSI-CVD). They had a two-year part-time training program in CVD, including education by cardiologists and experienced academic GPs in the field. They also have to pass an exam at the end of the training. In the Netherlands, there are in total 12 GPSI courses, including CVD, asthma and COPD, diabetes, elderly care, and palliative care. GPs may specialize in more than one expertise area. While it might be anticipated that GPSI-CVD perform better in their ‘domain’ than other GPs, this assumption lacks empirical evidence, and this hypothesis needs to be studied [13].

Clinical vignette surveys can illuminate physicians’ intended diagnostic strategies and reveal variations in diagnostic performance among physicians [14, 15].

We aimed to investigate whether GPSI-CVD more often considered HF in an older male patient who also had respiratory symptoms compared to other GPs. Assessing diagnostic knowledge and identifying potential knowledge gaps between GPSI-CVD and other GPs may be of help to set up training for GPs to increase awareness of HF and provide guidance on how to apply the diagnostic work-up in suspected HF cases.

## Methods

### Setting and participants

Two clinical vignettes were used (Table 1.). In August 2020, we approached all GPSI-CVD and GPSI-asthma/COPD in the Netherlands, and sampled GPs without specialization from the vicinity of Utrecht. In total 770 GPs were invited, including 80 GPSI-CVD and 690 other GPs not specialized in CVD. Participants received invitations via e-mail, and filling out the questions was possible by linkage to an online survey (see online Supplementary file). In the introduction, GPs were informed that the survey was about management of breathlessness and that clinical vignettes with multiple response options would be used to collect the data anonymously. GPs provided details on sex, years of working experience, practice size, and any non-CVD specializations. Participating GPs received 25 euros for survey completion.

**Table 1:** Vignettes presented to participating GPs and the evidence-based diagnostic work-up recommended by national and international guidelines

Vignette 1
A 72-year-old male person, known with a history of hypertension for 20 years and who smoked 30 packyears until the age of 60, visits the GP for a cold with a fever lasting for one week. He also reports shortness of breath, reduced exercise tolerance, restless sleep with nocturnal cough, and nocturia 2 to 3 times a night. On physical examination the apical impulse is broadened and sustained in left lateral decubitus position, and there are abnormal respiratory sounds over the basal fields of the lungs on both sides. Further physical examination is within normal limits.
Vignette 2
A 72-year old male person, known with a history of hypertension for 20 years, 30 packyears of smoking until the age of 60, and a history of COPD since 10 years for which using tiotropium and salmeterol inhalation medication, visits the GP for heavy coughing. He had a fever the last 3 days and is subfebrile (temperature 37.8 °C) at the morning of the visit. He has slightly yellowish discolored sputum, shortness of breath, reduced exercise tolerance, restless sleep with nocturnal cough and nocturia 2 to 3 times a night. On physical examination the apical impulse is broadened and sustained in left lateral decubitus position, and there are abnormal respiratory sounds over the basal fields of the lungs on both sides. Further physical examination is within normal limits.
What do the guidelines say?
According to existing clinical practice guidelines, the initial vignette should trigger the GP to consider three potential diagnoses or a combination of these: heart failure (HF), chronic obstructive pulmonary disease (COPD), and/or lower respiratory tract infection (LRTI). Long-term hypertension and smoking are significant risk factors for HF [9], while COPD should also be considered in patients above 40 years of age with a smoking history and with coughing, phlegm production, and/or progressive shortness of breath [16].Although both HF and COPD can be triggered by a LRTI, neither condition leads to fever on its own. Hence, a LRTI should also be considered as a ‘stand alone’ diagnosis in feverish patients, however, only having a cold is not likely to result in shortness of breath in patients unknown with a pulmonary or cardiac disease [17]. Restless sleep with nocturnal cough may occur in both HF and COPD, but these symptoms are more related to HF [1, 18]. Basal crackles are non-specific and can often be heard in HF, COPD, but also in restrictive pulmonary conditions including lung fibrosis and (bilateral) pneumonia [19, 20]. A broadened and sustained apical beat is a typical sign of HF [1, 9]. To rule out HF, natriuretic peptide measurement (amino-terminal pro-B-type natriuretic peptide (NT-proBNP) or B-type natriuretic peptide (BNP)) and electrocardiography (ECG) are recommended, followed by echocardiography if natriuretic peptides are above the exclusionary level and/or the electrocardiogram is abnormal [1, 9]. With spirometry COPD can be diagnosed, but it may overestimate its presence in patients with (unknown) HF if they have a pulmonary fluid overload which causes by external pressure on the alveoli and broncheoli an obstructive pattern [6, 21]. Finally, c-reactive protein (CRP) may be helpful to discriminate viral from bacterial LRTI. If a patient has a history of COPD as in the second vignette, HF, exacerbation of COPD and LRTI or a combination of these diagnoses may be suspected.

### Vignettes

GPs encountered two clinical scenarios of an older male person with hypertension reporting shortness of breath, reduced exercise tolerance, nocturnal cough, and nocturia. Physical examinations in both vignettes detected signs prompting HF considerations and additional tests, including a broadened and sustained apical impulse in the left decubital position and abnormal pulmonary breathing sounds (Table 1.).

In the first vignette, the 72-years old had a cold with fever, a longstanding smoking history, but no COPD.

The second vignette involved a similar 72-years old male person, however, now with a subfebrile temperature, and a history of COPD for which he used inhalation medication.

After each vignette, GPs answered the question, "What would you do?", selecting from a multiple-choice list (see online Supplementary file). GPs were allowed to select multiple answer options.

### Data analyses

Baseline characteristics were compared using independent t-test for continuous data and Pearson Chi-square or Fisher’s exact test for dichotomous data. GPs were categorized into GPSI-CVD or other GPs (i.e. GPs specialized in other diseases including COPD/astma, type 2 diabetes (T2D), or elderly care, and GPs without specialization). Answer options were divided into four categories according to the target diagnoses: (i) HF, (ii) COPD, (iii) exacerbation of COPD, or (iv) LRTI. Because combinations of these diagnoses could exist in both vignettes, GPs could choose multiple answer options, and ordering for tests could also be aimed at excluding a certain diagnosis. Thus, it is difficult to make an educated guess on what diagnosis the GP actually considered most likely in either vignette.

The answer option ‘other’ was manually categorized into one of the categories by two independent researchers, and in case of disagreement, they engaged in discussion to reach a final conclusion and if necessary a third researcher was asked. It was assumed that the answer option ‘chest X-ray’ was chosen if a LRTI was suspected because Dutch GP guidelines and European Society of Cardiology guidelines on HF discourage the use of chest X-ray for diagnosing HF or COPD [1, 9, 16].

According to existing evidence, we classified the answer options provided by the GPs as most likely targeted at diagnosing either of the four diagnoses. See Table 2. Data were analyzed using IBM SPSS Statistics 28.

**Table 2:** Answer options categorized as most likely targeted at diagnosing (confirming or excluding) either of the four most likely diagnoses to be considered based on the vignettes

Heart failure	COPD	Exacerbation of COPD	LRTI
Laboratory testing, including (NT-pro)BNP, Hb, Creatinin, Potassium	Spirometry	Start ipratropium bromide inhalation medication	Chest X-ray
12-lead resting electrocardiography	Start ipratropium bromide inhalation medication	Start prednisone	CRP measurement
Start furosemide 20–40 mg once daily	Referral to the pulmonologist	Start antibiotics and prednisone high dose	Start antibiotics
Referral to the cardiologist		Referral to the pulmonologist	

*Abbreviations*: *COPD *Chronic obstructive pulmonary disease, *LRTI *Lower respiratory tract infection; (NT-pro)BNP, (N-terminal pro) b-type natriuretic peptide, *Hb *Hemoglobin

## Results

### Respondents

In total, 148 GPs (19.2%) completed the questionnaire; 25 (31.3%) GPSI-CVD and 123 (17.8%) other GPs not specialized in CVD. The latter group included 107 GPs without specialization and 16 GPs with special interests in other diseases; eight in COPD/asthma, three in T2D and five in elderly care. GPSI-CVD were more often male and had on average more years of working experience. See Table 3.

**Table 3:** Baseline characteristics of participating GPs

	GPSI-CVD (*n* = 25)	Other GPs^a^ (*n* = 123)	*p*-value
Male sex	16 (64.0)	44 (35.8)	0.009^a^
Years of working experience as a GP	18.9 ± 7.5	13.0 ± 9.5	0.002^a^
Years of working experience as a GPSI-CVD	6.2 ± 3.2	-	-
Patients enlisted in practice	2,470 ± 899.3	2,487 ± 1525.3	0.946

*Abbreviations*: *GP *General practitioner, *GPSI-CVD *GP with special interest in cardiovascular disease

Data presented as mean ± standard deviation or count (%)

^a^This group included 107 GPs without specialization and 16 GPs specialized in other diseases; eight in COPD/asthma, three in type 2 diabetes and five in elderly care

### Vignettes

In vignette 1, GPSI-CVD compared to other GPs more likely considered ordering ECG (64.0% vs. 32.5%, *p* = 0.003). Other tests focused on HF tended to be more considered by GPSI-CVD than other GPs; natriuretic peptide testing 84.0% vs. 75.6% and prescribing furosemide 40.0% vs. 30.9%. Spirometry (24.0% vs. 17.1%), prescribing inhalation medication (4.0% vs. 9.8%), chest X-ray (40.0% vs 30.1%), and CRP measurement (72.0 vs. 77.2%) were considered similarly by both groups of GPs. See Table 4.

**Table 4:** Overview of answers given by GPSI-CVD and other GPs as a response to the two vignettes

Answer option	GPSI-CVD (*n* = 25)	Other GPs^a^ (*n* = 123)	*p*-value
Vignette 1			
‘Wait and see’ for 2 weeks	0	2 (1.6)	1.000
Lab including (NT-pro)BNP, Hb, Creatinine, Potassium	21 (84.0)	93 (75.6)	0.363
12-lead resting electrocardiography	16 (64.0)	40 (32.5)	0.003^a^
Start furosemide 20–40 mg once daily	10 (40.0)	38 (30.9)	0.375
Referral to cardiologist	0	0	-
Spirometry	6 (24.0)	21 (17.1)	0.414
Start ipratropium bromide inhalation medication	1 (4.0)	12 (9.8)	0.354
Referral to pulmonologist	0	0	-
Chest X-ray	10 (40.0)	37 (30.1)	0.331
CRP measurement	18 (72.0)	95 (77.2)	0.574
Vignette 2			
Lab including (NT-pro)BNP, Hb, Creatinine, Potassium	14 (56.0)	40 (32.5)	0.026^a^
12-lead resting electrocardiography	9 (36.0)	15 (12.2)	0.003^a^
Start furosemide 20-40mg once daily	3 (12.0)	17 (13.8)	0.808
Referral to cardiologist	0	0	-
Spirometry	0	0	-
Start ipratropium bromide inhalation medication	1 (4.0)	8 (6.5)	0.633
Start prednisone high dose	8 (32.0)	46 (37.4)	0.609
Start antibiotics and prednisolone high dose	6 (24.0)	34 (27.6)	0.709
Referral to pulmonologist	0	0	-
Chest X-ray	7 (28.0)	13 (10.6)	0.020^a^
CRP measurement	20 (80.0)	81 (65.9)	0.166
Start antibiotics	4 (16.0)	15 (12.2)	0.604

*Abbreviations*: *GP *General practitioner, *GPSI-CVD *GP with special interest in cardiovascular disease, *COPD *Chronic obstructive pulmonary disease; (NT-pro)BNP, (N-terminal pro) b-type natriuretic peptide, *Hb *Hemoglobin

Data are presented as absolute numbers and percentages (%)

^a^This group included 107 GPs without specialization and 16 GPs specialized in other diseases; eight in COPD/asthma, three in type 2 diabetes and five in elderly care

In vignette 2, GPSI-CVD more often considered ordering ECG (36.0% vs. 12.2%, *p* = 0.003), natriuretic peptide testing (56.0% vs. 32.5%, *p* = 0.026), and chest X-ray (28.0% vs. 10.6%, *p* = 0.020) than other GPs. Prescribing furosemide (12.0% vs. 13.8%), inhalation medication (4.0% vs. 6.5%), and CRP measurement (80.0% vs. 65.9%) were considered similarly by both groups of GPs. See Table 4.

Most GPs, both GPSI-CVD and other GPs, seemed to consider multiple diagnoses in both vignettes given the investigations they filled out to initiate. In vignette 1, 84.0% vs. 78.9% of the GPSI-CVD and other GPs, respectively, and in vignette 2, 56.0% vs. 36.6%, filled out at least one item out of (i) laboratory testing including natriuretic peptide, (ii) ECG, (iii) prescription of furosemide, and (iv) referral to the cardiologist. See Table 5.

**Table 5:** Most likely disease considered by GPSI-CVD and other GPs based on the answers reported in Table 3 and the categorization in Table 4

Disease likely considered	GPSI-CVD (*n* = 25)	Other GPs^a^ (*n* = 123)	*p*-value
**Vignette 1**			
Heart failure	21 (84.0)	97 (78.9)	0.560
COPD	6 (24.0)	29 (23.6)	0.964
LRTI	21 (84.0)	108 (87.8)	0.604
**Vignette 2**			
Heart failure	14 (56.0)	45 (36.6)	0.071
Exacerbation of COPD	17 (68.0)	90 (73.2)	0.598
LRTI	24 (96.0)	111 (90.2)	0.354

*Abbreviations*: *GP *General practitioner, *GPSI-CVD *GP with special interest in cardiovascular disease, *COPD *Chronic obstructive pulmonary disease, *LRTI *Lower respiratory tract infection

Data are presented as absolute numbers and percentages (%)

^a^This group included 107 GPs without specialization and 16 GPs specialized in other diseases; eight in COPD/asthma, three in type 2 diabetes and five in elderly care

## Discussion

### Main findings

When presented with a vignette of an older male person, who has shortness of breath, reduced exercise tolerance and other symptoms and signs suggestive of HF along with a cold and a fever, GPSI-CVD more often ordered ECG (64.0% vs. 32.5%, *p* = 0.003) compared to other GPs. When presented with a vignette of an older male person already diagnosed with COPD who has a subfebrile temperature after a period of fever, GPSI-CVD were also more inclined to order ECG (36% vs 12%, *p* = 0.003), but now also (NT-pro)BNP (56% vs 33%, *p* = 0.026) compared to other GPs, as recommended in HF guidelines. However, when comparing GPSI-CVD to other GPs for considering one item out of (i) laboratory testing including natriuretic peptide, (ii) ECG, (iii) prescription of furosemide, and (iv) referral to the cardiologist, there was no significant difference between the two groups of GPs; in vignette 1, 84.0% vs. 78.9% and in vignette 2, 56.0% vs. 36.6%, respectively.

The specialization of GPSI-CVD does not appear to have resulted in a lack of attention towards other diseases, particularly respiratory diseases. The vast majority of GPs, both GPSI-CVD and other GPs, seem to consider multiple diagnoses in both vignettes, which seems adequate given that multiple diseases could cause the symptoms and signs presented in both vignettes. This finding aligns with the understanding that LRTI can either be an alternative diagnosis for HF or COPD, or it can go along with either condition [22, 23]. Similarly, COPD might be an alternative diagnosis to consider alongside HF, but it can also concurrently exist with HF [5–8].

### Strengths and limitations

Our study is unique in that it is the first that assessed whether GPSI-CVD perform more investigations aimed at diagnosing HF than other GPs in an older male person with both respiratory and suggestive HF symptoms. A common diagnostic dilemma GPs encounter in everyday practice. Clinical vignettes are a valuable method in providing insight into physician’s diagnostic reasoning.

Our study has a number of limitations. First, a clinical vignette study does not fully capture the intricacies of an actual patient encounter. The structured nature of the survey and the format of multiple-choice answers has impact on GPs’ decisions, which potentially deviates from what they would actually do in everyday practice. This could have resulted in an overestimation of GPs’ use of additional diagnostic tests beyond history taking and physical examination, importantly, however, similarly for both groups, thus very unlikely affecting the comparison we studied. A second limitation is that we focus on a common diagnostic dilemma; an older person with both respiratory and suggestive HF symptoms, thus not covering the complete spectrum of diagnosing HF in general practice. Third, we have a modest response rate to the online survey, although, not different from literature [24, 25]. This may have induced selection of more motivated GPs, however, in both groups, thus not resulting in bias of the comparison between GPSI-CVD and other GPs as we did in our study. Fourth, the multiple-choice options focused on the most likely diagnoses to be considered (HF, COPD or exacerbation of COPD, LRTI), not on all potential over 30 causes of shortness of breath [26]. Finally, the two vignettes were not presented at random to the GPs. This might have caused ‘carry-over effects’ that could affect the subsequent responses of GPs. Importantly, however, this shortcoming was consistent in both groups and thus likely did not affect the comparison between the two groups of GPs.

### Insights from the literature

Up to 20% of patients with COPD aged 65 years or over may have concurrent HF, which is approximately 3 times higher than in the general population [5, 27, 28]. COPD and HF share common risk factors, such as smoking and low socio-economic status [29]. They share pathophysiological mechanisms, e.g. low grade systemic inflammation, but also symptoms and signs, e.g., shortness of breath, reduced exercise tolerance, nightly cough and crackles on auscultation of the lungs [3, 29–31]. Discriminating HF from COPD is hampered also because patients with unrecognized HF may have an obstructive pattern with spirometry due to pulmonary fluid overload [6, 21]. Misclassification of COPD in patients with HF is therefore high in both primary care and hospital setting if only based on spirometry, without bodybox measurements considering the ratio of residual volume and total lung capacity (RV/TLC ratio) [21, 32]. Independent clinical predictors of HF among patients labelled with COPD are a history of ischaemic heart disease, a high body mass index, raised heart rate, but also a laterally displaced apical beat [12]. Considering these parameters might assist GPs in deciding the need for additional diagnostic testing for HF in individual patients known to have stable COPD. But just natriuretic peptide testing could already help assist identifying HF in patients with COPD applying the same exclusionary cut-point as generally recommended in the HF guidelines [12].

### Implications for clinical practice

GPSI-CVD, with their specialized training in cardiovascular medicine, might be more attentive to symptoms indicating cardiovascular disease, including HF. Their familiarity with HF guidelines may make them more likely to consider HF when confronted with patients with symptoms suggestive of HF, also if these patients have respiratory symptoms due to either a concurrent LRTI or history of COPD. Given the high prevalence of unrecognized HF in patients labelled with COPD, we suggest conducting yearly natriuretic peptide testing in such individuals.

GPSI-CVD not only more often request ECG, possibly due to their additional training in ECG interpretation, and natriuretic peptide tests, but they also seem to consider multiple diagnostic tests targeted at diagnosing HF more often than other GPs. GPSI-CVD are in the position to actively provide training for other GPs and practice nurses and thus can contribute to increasing the awareness of HF and the diagnostic steps in the work-up in primary care. Both awareness and improved diagnostic knowledge can on their turn boost the management of patients with a definite diagnosis of HF, preferably cooperatively with cardiologists and HF nurses.

## Conclusions

Most GPs seemed to consider multiple diagnoses, including HF, with GPSI-CVD more likely performing ECG and natriuretic peptide testing if an older male person has both respiratory and suggestive HF symptoms. This suggests GPSI-CVD adhere more closely to the diagnostic paragraph in HF guidelines than other GPs, as illustrated by their responses to the two presented clinical scenarios. Further training of GPs in diagnostic procedures to be considered in older people with shortness of breath could help to uncover previously unrecognized HF.

### Supplementary Information

Supplementary Material 1.

## Data Availability

All data generated or analysed during this study are included in this published article.

## Abbreviations

BNP: Brain natriuretic peptide
CRP: C-reactive protein
COPD: Chronic obstructive pulmonary disease
ECG: Electrocardiography
GP: General practitioner
GPSI-CVD: General practitioner specialized in cardiovascular disease
HF: Heart failure
LRTI: Lower respiratory tract infection
NT-proBNP: Amino-terminal pro-B-type natriuretic peptide
T2D: Type 2 diabetes
