# Supplementary material for: Comparing the diagnostic considerations between general practitioners with a special interest in cardiovascular disease and those without in patients with symptoms suggestive of heart failure: a vignette study
Source: BMC Prim Care. 2024 Jun 14;25:216. doi: 10.1186/s12875-024-02466-6 (PMC11177529; doi:10.1186/s12875-024-02466-6)
Supplement: Supplementary file 1 — Supplementary Material 1. [file 12875_2024_2466_MOESM1_ESM.docx]

**Supplementary file: Online survey**

**Management of breathlessness by the general practitioner**

Dear colleague,

We hope you want to fill out this survey and help us to provide insight into the management of breathlessness in primary care. Clinical vignettes with multiple response options will be used. For completing the questionnaire we offer you a voucher of 25 euros as compensation for your time. Data will be collected anonymously.

Kind regards,

Frans Rutten (Julius Centre for Health Sciences and Primary Care, UMC Utrecht)

Monika Hollander (Leidsche Rijn Julius Healthcare Centers & Julius Centre for Health Sciences and Primary Care, UMC Utrecht)

Ingrid Looijmans (Leidsche Rijn Julius Healthcare Centers & Julius Centre for Health Sciences and Primary Care, UMC Utrecht)

1. **What is your sex?**

🞎 Male

🞎 Female

1. **How many years of working experience do you have?**

__________ years

1. **What is the size of your practice?**

__________ patients

1. **If you are a general practitioner specialized in a clinical domain, could you please specify your area of expertise?**

____________________

**We would like to present you with two vignettes.**

**Vignette 1:**

A 72-year-old male person, known with a history of hypertension for 20 years and who smoked 30 packyears until the age of 60, visits the GP for a cold with a fever lasting for one week. He also reports shortness of breath, reduced exercise tolerance, restless sleep with nocturnal cough, and nocturia 2 to 3 times a night.

On physical examination the apical impulse is broadened and sustained in left lateral decubitus position, and there are abnormal respiratory sounds over the basal fields of the lungs on both sides. Further physical examination is within normal limits.

What would you do? (Multiple options possible)

🞎 ‘Wait and see’ for 2 weeks; See whether the symptoms persist

🞎 I order laboratory testing, including (NT-pro)BNP, Hb, Creatinine and Potassium

🞎 I order a 12-channel ECG or perform one

🞎 I order a chest X-ray

🞎 I order spirometry

🞎 I prescribe furosemide 20-40 mg once daily

🞎 I prescribe ipratropium bromide inhalation medication

🞎 I refer to the pulmonologist without additional diagnostic testing

🞎 I refer to the cardiologist without additional diagnostic testing

🞎 I determine CRP

🞎 Other (please provide further explanation)

**Vignette 2:**

A 72-year old male person, known with a history of hypertension for 20 years, 30 packyears of smoking until the age of 60, and a history of COPD since 10 years for which using tiotropium and salmeterol inhalation medication, visits the GP for heavy coughing. He had a fever the last 3 days and is subfebrile (temperature 37.8 °C) at the morning of the visit. He has slightly yellowish discolored sputum, shortness of breath, reduced exercise tolerance, restless sleep with nocturnal cough and nocturia 2 to 3 times a night.

On physical examination the apical impulse is broadened and sustained in left lateral decubitus position, and there are abnormal respiratory sounds over the basal fields of the lungs on both sides. Further physical examination is within normal limits.

What would you do? (Multiple options possible)

🞎 I prescribe antibiotics

🞎 I prescribe prednisolone high dose

🞎 I prescribe both antibiotics and prednisolone high dose

🞎 I order laboratory testing, including (NT-pro)BNP, Hb, Creatinine and Potassium

🞎 I order a 12-lead channel ECG or perform one

🞎 I order a chest X-ray

🞎 I order spirometry

🞎 I prescribe furosemide 20-40 mg once daily

🞎 I prescribe ipratropium bromide inhalation medication

🞎 I refer to the pulmonologist without additional diagnostic testing

🞎 I refer to the cardiologist without additional diagnostic testing

🞎 I determine CRP

🞎 Other (please provide further explanation)
